# Cell Adhesion Molecule Close Homolog of L1 (CHL1) Guides the Regrowth of Regenerating Motor Axons and Regulates Synaptic Coverage of Motor Neurons

**DOI:** 10.3389/fnmol.2018.00174

**Published:** 2018-05-24

**Authors:** Daria Guseva, Igor Jakovcevski, Andrey Irintchev, Iryna Leshchyns’ka, Vladimir Sytnyk, Evgeni Ponimaskin, Melitta Schachner

**Affiliations:** ^1^Zentrum für Molekulare Neurobiologie Hamburg, University Hospital Hamburg-Eppendorf, Hamburg, Germany; ^2^Department of Cellular Neurophysiology, Hannover Medical School, Hannover, Germany; ^3^Department of Experimental Neurophysiology, German Center for Neurodegenerative Diseases (DZNE), Bonn, Germany; ^4^Department of Otorhinolaryngology, Jena University Hospital, Jena, Germany; ^5^School of Biotechnology and Biomolecular Sciences, South Western Sydney Clinical School, The University of New South Wales, Sydney, NSW, Australia; ^6^Department of Cell Biology and Neuroscience, W. M. Keck Center for Collaborative Neuroscience, Rutgers University, Piscataway, NJ, United States; ^7^Center for Neuroscience, Shantou University Medical College, Shantou, China

**Keywords:** cell adhesion molecule close homolog of L1 (CHL1), femoral nerve, peripheral nerve regeneration, motor neuron, mouse, preferential motor re-innervation

## Abstract

The close homolog of L1 (CHL1) is a cell adhesion molecule involved in regulation of neuronal differentiation and survival, neurite outgrowth and axon guidance during development. In the mature nervous system, CHL1 regulates synaptic activity and plasticity. The aim of the present study was to evaluate the influence of CHL1 on peripheral nerve regeneration after trauma. Using the established model of mouse femoral nerve regeneration, CHL1 knock-out mice were investigated in comparison to the wild type littermates. First, non-injured mice of both genotypes were compared regarding the synaptic phenotypes in the corresponding spinal cord segment. While no differences in phenotypes were detectable in the femoral nerve, corresponding segments in the spinal cord were observed to differ in that inhibitory perisomatic innervation of motor neurons was increased in CHL1-deficient mice, and numbers of perisomatic cholinergic synapses on motor neuronal somata were reduced. Regarding the femoral nerve after injury, CHL1-deficient mice demonstrated preferential motor axon regrowth into the saphenous vs. quadriceps branch after nerve transection upstream of the nerve bifurcation by 8 weeks after transection, indicating decreased preferential motor re-innervation. Furthermore, in injured wild-type mice, enhanced CHL1 expression was observed in regenerating axons in the proximal nerve stump upstream of the bifurcation at days 1, 3, 5, 7 and 14, and in the distal stump at days 7 and 14 after injury, when compared to non-injured mice. Injury-related upregulation of CHL1 expression was more pronounced in axons than in Schwann cells. Despite a more pronounced capacity for preferential motor axon regrowth in wild-type vs. mutant mice, only a tendency for difference in recovery of motor functions was observed between genotypes, without statistical significance Taken together, these results indicate that CHL1 is involved in peripheral nerve regeneration, because it guides regrowing axons into the appropriate nerve branch and regulates synaptic coverage in the spinal cord.

## Introduction

The close homolog of L1 (CHL1) is a cell adhesion molecule of the immunoglobulin superfamily expressed at the cell surface of neurons and glial cells in the central (CNS) and peripheral nervous system (PNS; Hillenbrand et al., 1999; Rolf et al., 2003). In the developing nervous system, CHL1 plays a role in regulation of neuronal differentiation and survival, neurite outgrowth, and axon guidance (Chen et al., 1999; Montag-Sallaz et al., 2002; Nishimune et al., 2005; Tian et al., 2012; Katic et al., 2014; Barão et al., 2015; Schmalbach et al., 2015). In the mature nervous system, CHL1 is involved in regulation of synaptic activity and plasticity, including learning and memory (Leshchyns’ka et al., 2006; Morellini et al., 2007; Kolata et al., 2008; Pratte and Jamon, 2009; Andreyeva et al., 2010). In humans, single nucleotide polymorphisms, deletions and duplications in CHL1 gene are associated with schizophrenia, autism, epilepsy and intellectual disability (Sakurai et al., 2002; Chen et al., 2005; Salyakina et al., 2011; Shoukier et al., 2013; Palumbo et al., 2015; Li et al., 2016).

The extracellular domain of CHL1 mediates homophilic (CHL1-CHL1) adhesion (Jakovcevski et al., 2007) as well as heterophilic interaction with other cell adhesion molecules, including integrins, NB-3, semaphorin 3A, neuropilin 1 (Wright et al., 2007; Barão et al., 2015), and vitronectin, as well as the protease-related plasminogen activator inhibitor-2 (Buhusi et al., 2003; Ye et al., 2008; Katic et al., 2014). The intracellular domain of CHL1 links it to other cell surface receptors, such as the serotonin 2c receptor (Kleene et al., 2015), and scaffolding proteins, such as disrupted-in-schizophrenia 1 (DISC1; Ren et al., 2016).

While the heterophilic interactions of CHL1 promote neurite outgrowth (Hillenbrand et al., 1999; Katic et al., 2014), the homophilic interactions of CHL1 inhibit neurite outgrowth of neurons from the developing CNS (Jakovcevski et al., 2007; Wu et al., 2010; Katic et al., 2014). CHL1 and particularly its proteolytic fragment generated by BACE1-mediated cleavage, is required for Sema3A-induced growth cone collapse in thalamic and hippocampal neurons (Barão et al., 2015), and CHL1 deletion in mice selectively disrupts the projection of somatosensory thalamic axons from the ventrobasal nuclei (Wright et al., 2007). CHL1 expression is induced in neurons of the CNS and PNS that are capable of axonal regrowth after injury (Chaisuksunt et al., 2000, 2003; Zhang et al., 2000). After spinal cord injury, CHL1 is overexpressed by glial scar forming astrocytes, and homophilic interactions with CHL1 on severed axons inhibit axonal regrowth and impair locomotor recovery (Jakovcevski et al., 2007; Wu et al., 2011). CHL1 enhances the expression of NB-3 (Ye et al., 2008) and is involved in NB-3-mediated inhibition of axonal regeneration after spinal cord injury via receptor protein tyrosine phosphatase σ (RPTPσ)-dependent downregulation of mammalian target of rapamycin (mTOR) activity in neurons (Huang et al., 2016).

CHL1 is expressed in the femoral and sciatic nerves of newborn mice (Hillenbrand et al., 1999), but in the adult it is unknown whether CHL1 is functionally involved in peripheral nerve regeneration. In the present study, we used the established femoral nerve injury model in mice, and analyzed CHL1 expression in the nerve, as well as locomotor recovery, numbers of regenerating axons, axonal targeting, expression by Schwann cells, and perisomatic innervation of femoral motor neurons. Our results indicate that CHL1 is involved in targeting of regenerating axons and synaptic plasticity after mouse femoral nerve injury.

## Materials and Methods

### Animals

Ten- to 12-week-old CHL1-deficient (CHL1^−/−^) female mice and age-matched wild-type (CHL1^+/+^) littermates from heterozygous breeding on a mixed C57BL/6J-129Ola genetic background (Montag-Sallaz et al., 2002) had been backcrossed onto the C57BL/6J background for more than eight generations. Animals were bred and maintained at the Universitätsklinikum Hamburg-Eppendorf. Genotyping of the mice from the CHL1 stock was performed as described (Montag-Sallaz et al., 2002). Mice were kept under specific pathogen conditions with food and tap water *ad libitum*, and an artificial 12 h light/dark cycle. All animal experiments were approved by the local authorities of the State of Hamburg (#098/09) and conformed to the guidelines set by the European Union. Numbers of animals studied in different experimental groups and at different time periods after surgery are indicated in the text and figure legends. All animal treatments, data acquisition and analyses were performed in a blinded manner.

### Experimental Design

In our experiments, we have taken an advantage of a valuable paradigm of the femoral nerve injury model in rodents, which allows studies on accuracy of axonal pathway finding and molecular mechanisms determining re-innervation selectivity (Brushart, 1988, 1993; Madison et al., 1996; Simova et al., 2006). The femoral nerve bifurcates into two major branches: a quadriceps muscle branch containing motor and sensory axons, and a purely sensory branch, the saphenous branch, innervating the skin. After lesion of the common nerve trunk, motor axons initially regrow at random into both the quadriceps branch, and the saphenous branch. After the proceeding of re-innervation, the number of motor neurons correctly projecting into the quadriceps branch increases, a phenomenon known as preferential motor re-innervation (Brushart, 1988, 1993). Experiments were performed as described (Guseva et al., 2009). Two sets of experiments were carried out: in Experiment 1, we analyzed functional recovery in female CHL1^−/−^ and CHL1^+/+^ mice during a recovery period of 8 weeks following femoral nerve injury. Subsequently, these animals were subjected to retrograde labeling of regenerated motor neurons and, following a survival period of 1 week, injured and intact contralateral femoral nerves from one mouse were sampled for histological analyses. Non-injured, age- and sex-matched mice of both genotypes were also analyzed. These mice were subjected only to retrograde tracing and used for morphological analysis of quadriceps motor neurons. In Experiment 2, the femoral nerves of CHL1^−/−^ and CHL1^+/+^ mice were transected, and 1, 3, 5, 7 and 14 days thereafter the proximal and distal nerve segments were harvested for analyses of CHL1 expression.

### Transection and Surgical Repair of the Femoral Nerve

Surgery was performed as described (Simova et al., 2006). Briefly, mice were anesthetized by intraperitoneal injections of 0.4 mg/kg fentanyl (Fentanyl-Janssen, Janssen, Neuss, Germany), 10 mg/kg droperidol (Dehydrobenzperidol, Janssen) and 5 mg/kg diazepam (Valium 10 Roche, Hoffman—La Roche, Grenzach-Wyhlen, Germany). The right femoral nerve was exposed and transected approximately 3 mm proximal to the bifurcation of the saphenous and quadriceps branches. The cut ends of the nerve were inserted into a polyethylene tube (3 mm length, 0.58 mm inner diameter, Becton Dickinson, Heidelberg, Germany) and fixed with a single epineural 11–0 nylon stitch (Ethicon, Norderstedt, Germany) so that a 2 mm gap was present between the proximal and distal nerve stumps. The tube was filled with phosphate buffered saline (PBS, pH 7.4) and the skin was closed with 6–0 sutures (Ethicon). To prevent hypothermia, the mice were then kept in a warm room (35°C) until full recovery from anesthesia.

### Analysis of Motor Function

Analysis of the motor function was performed over a time-period of 8 weeks using a quantitative video-based single-frame motion analysis (Irintchev et al., 2005b). In this test, the animal walks voluntarily from one end of a horizontal beam (length 1000 mm, width 40 mm) towards its home cage located at the other end of the beam. To evaluate locomotor function, mice were accustomed, in 3–4 trials, to beam-walking prior to surgery. For all mice, a rear view of one walking trial was captured once before surgery and then at different time-points after surgery with a high-speed camera (A602fc, Basler, Ahrensburg, Germany) at 100 frames per second and stored on a personal computer in Audio Video Interleaved (AVI) format. The video sequences were examined using SIMI-Motion 7.0 software (SIMI Reality Motion Systems, Unterschleissheim, Germany). Selected frames in which the animals were seen in defined phases of the step cycle were used for measurements of two parameters: the heels-tail angle (HTA) and the foot-base angle (FBA) as described (Irintchev et al., 2005b). Both parameters are directly related to the ability of the quadriceps muscle to keep the knee joint extended during contralateral swing phases. As a relative measure of functional recovery at different time-points after nerve injury, we calculated the stance recovery index (RI), which is a mean of the RI for the HTA and the FBA. The index for each angle is calculated in percent as:

$$RI\;=\;\left[\left(X_{\text{reinn}}\;-\;X_{\text{den}})/(X_{\text{pre}}\;-\;X_{\text{den}}\right)\right]\times 100$$

where *X*_pre_, *X*_den_ and *X*_reinn_ are values prior to operation, during the state of denervation (7 days after injury) and at different time-points after injury.

### Retrograde Labeling of Motor Neurons

At 8 weeks after nerve transection, animals were anesthetized with fentanyl, droperidol and diazepam for retrograde labeling of regenerated motor neurons (Simova et al., 2006). After exposure of the right femoral nerve, a piece of Parafilm (Pechiney Plastic Packaging, Chicago, IL, USA) was inserted underneath the nerve and the two nerve branches were transected approximately 5 mm distal to the bifurcation. Fluorescence retrograde tracers were applied to the cut nerve ends in powder form: Fluoro-ruby (tetramethylrhodamine dextran, Molecular Probes, Leiden, Netherlands) to the saphenous (cutaneous) branch (exclusively sensory branch) and Fast Blue (EMS-Chemie, Großumstadt, Germany) to the quadriceps (muscle) branch (mixed nerve containing sensory and motor axons). Thirty minutes after dye application, the nerve stumps were rinsed with PBS and the wound was closed. This labeling procedure allows visualization of all femoral motor neurons that have successfully regrown their axons beyond the lesion site. In addition, it is possible to differentiate between motor neurons with axons correctly projecting into the quadriceps branch and aberrantly projecting motor neurons sending axons to the saphenous branch only or to both the saphenous and the quadriceps branch. The same labeling procedure applied to non-injured CHL1^−/−^ and CHL1^+/+^ mice, i.e., mice which had not been subjected to nerve transection and repair, allows to estimate the normal number of quadriceps motor neurons all of which project to the quadriceps branch only.

### Preparation of Tissue for Morphological Analyses

One week after retrograde labeling, mice were anesthetized by intraperitoneal injection of 16% sodium pentobarbital solution (Narcoren^®^, Merial, Hallbergmoos, Germany, 5 μl/g body weight) and were transcardially perfused with 4% formaldehyde in 0.1 M sodium cacodylate buffer, pH 7.3. The lumbar spinal cord was removed, post-fixed overnight at 4°C and then immersed in 15% sucrose solution in 0.1 M cacodylate buffer, pH 7.3, for 1 day at 4°C. Afterwards, the tissue was frozen for 2 min in 2-methyl-butane (Isopentane, Carl Roth, Karlsruhe, Germany) precooled to −30°C. For sectioning, the spinal cord segment was attached to a cryostat specimen holder using TissueTek^®^ (Sakura Finetek Europe, Zoeterwoude, Netherlands). Serial transverse sections of 25 μm thickness were cut on a cryostat (Leica CM3050, Leica Instruments, Nußloch, Germany) and transferred to SuperFrost^®^Plus glass slides (Roth, Karlsruhe, Germany). The sections were air-dried for at least 1 h at room temperature (RT) and mounted in anti-quenching medium (Fluoromount G, Southern Biotechnology Associates, Biozol, Eching, Germany). These sections were used for counting retrogradely labeled motor neurons.

Both injured and intact contralateral femoral nerves were dissected from perfusion-fixed animals. The tissue samples were post-fixed in 1% osmium tetroxide (Polysciences Europe, Eppelheim, Germany) in 0.1 M sodium cacodylate buffer, pH 7.3, for 1 h at RT, dehydrated and embedded in resin according to standard protocols. One micrometer-thick cross-sections from the quadriceps and saphenous nerve branches were cut at a distance of approximately 3 mm distal to the bifurcation and stained with 1% toluidine blue/1% borax in distilled water for analysis of axon numbers in regenerated and intact nerve branches.

### Counting of Retrogradely Labeled Motor Neurons

Analysis was performed using a fluorescence microscope (Axiophot 2, Zeiss, Oberkochen, Germany) with appropriate filter sets. Each section, containing typically 2–5 labeled cell profiles, was examined using a 40× objective by focusing through the section thickness starting from the top surface. All profiles, except those visible at the top surfaces of sections, were counted (Simova et al., 2006). The application of this simple stereological principle prevented double counting of labeled cells and allowed precise evaluation of cell numbers.

### Immunofluorescence Staining of Perisomatic Axon Terminals

Immunofluorescence staining was performed as described (Irintchev et al., 2005a) using commercial antibodies at optimal dilutions (Table 1). Sections of spinal cord containing retrogradely labeled motor neurons were freed from coverslips and mounting medium by soaking in PBS. Antigen retrieval was performed by immersion into 0.01 M sodium citrate solution, pH 9.0, heated to 70°C in a water bath for 30 min. Blocking of non-specific binding sites was then performed using PBS containing 0.2% Triton X-100 (Sigma), 0.02% sodium azide (Merck, Darmstadt, Germany) and 5% normal goat or normal donkey serum for 1 h at RT. Incubation with primary antibodies against vesicular GABA transporter (VGAT) or choline acetyltransferase (ChAT; Table 1), diluted in PBS containing 0.5% lambda-carrageenan (Sigma) and 0.02% sodium azide, was carried out at 4°C for 3 days. For a given antigen, all sections were stained in the same solution kept in screw-capped staining plastic jars (capacity 35 ml, 10 slides, Roth). After washing in PBS, appropriate Cy3-conjugated secondary antibodies (Jackson ImmunoResearch Europe, Suffolk, UK) diluted 1:200 in PBS-carrageenan solution were applied for 2 h at RT. After a subsequent washing in PBS, cell nuclei were stained for 10 min at RT with *bis*-benzimide solution (Hoechst 33258 dye, 5 μg/ml in PBS, Sigma) and the sections were mounted in anti-quenching medium. Specificity of staining was controlled by omitting the primary antibody or replacing it by an equivalent amount of non-immune IgG or serum derived from the same species as the specific antibody. These controls did not show labeling.

**Table 1:** Primary antibodies used in this study.

Antibody	Cellular phenotypes/structures recognized	Host	Code/clone	Dilution	Source
S100	Schwann cells	Rabbit	Z0311	1:15,000	DakoCytomation, Hamburg, Germany
βIII-tubulin	Axons	Rabbit	PRB-435P-100	1:2000	Covance, Berkeley, CA, USA
CHL1	Neurons and Schwann cells	Goat	AF2147	1:100	R&D Systems, Wiesbaden-Nordenstadt, Germany
L1 Ab 557	Neurons	Rat		1:500	Kadmon et al. (1990)
Vesicular GABA transporter (VGAT)	Inhibitory (GABA- and glycinergic) synaptic terminals	Mouse	131 011	1:1000	Synaptic Systems, Göttingen, Germany
Choline acetyltransferase (ChAT)	Cholinergic synaptic terminals	Goat	AB144P	1:100	Chemicon

### Analyses of Perisomatic Synaptic Terminals

Sections containing retrogradely labeled motor neurons and additionally stained for ChAT and VGAT were used to estimate motor neuron perisomatic coverage (Apostolova et al., 2006; Simova et al., 2006). Density (number per unit length) of perisomatic terminals was estimated for motor neurons that correctly projected to the quadriceps nerve branch of the femoral nerve (identified by Fast Blue back-labeling). Stacks of images of 1 μm thickness were obtained on a confocal microscope (Leica DM IRB, Leica, Wetzlar, Germany) using a 63× oil immersion objective and digital resolution of 1024 × 1024 pixels. One image per cell at the level of the largest cell body cross-sectional area was used to measure number of perisomatic puncta. These measurements were performed using the Image Tool 2.0 software program (University of Texas, San Antonio, TX, USA).

### Analyses of Axons in the Regenerated Nerve Branches

Total numbers of myelinated axons per nerve cross-section were estimated on an Axioskop microscope (Zeiss) equipped with a motorized stage and Neurolucida software-controlled computer system (MicroBrightField Europe, Magdeburg, Germany) using a 100× oil objective (Simova et al., 2006).

### Analysis of CHL1 Expression During Femoral Nerve Regeneration

Femoral nerve surgery was performed as described above, avoiding both a 2 mm gap, the use of polyethylene tubes, and suture. We decided to apply this experimental design in order to allow the motor and sensory axons to regenerate in their native environment during a short time frame (1, 3, 5, 7 or 14 days) with as little intervention as possible. The mice (*n* = 2–3 per genotype and time-point) were perfused 1, 3, 5, 7 or 14 days after injury and femoral nerve segments approximately 15 mm in length, including the proximal and distal stumps, were post-fixed and cryoprotected by sucrose infiltration as described above. For indirect immunofluorescence, the tissue samples were immersed in TissueTek^®^ medium and frozen in liquid nitrogen. Longitudinal (5-μm-thick) cryostat sections were used in double-labeling immunofluorescence experiments using antibodies listed in Table 1. Indirect immunofluorescence was performed as described above for perisomatic terminals using the primary antibodies alone for single labeling or mixed at optimal dilutions for double labeling. Appropriate Alexa594- and Alexa488-conjugated antibodies pre-absorbed with normal sera from diverse species to prevent cross-reactions (Multiple Labeling antibodies, Jackson ImmunoResearch) were used in double labeling experiments. Specificity of staining was controlled by omitting the primary antibody or replacing it by an equivalent amount of non-immune IgG or serum. These controls did not show any staining. For the analysis of co-localization of CHL1 and S100 (a Schwann cell marker), and CHL1 and βIII-tubulin (neuronal marker), we used Pearson’s correlation coefficient, which is the co-localization coefficient to express the intensity correlation of co-localizing objects in each component of a dual-color image. The quantification of co-localization between two fluorescence channels in this case considers the presence of both fluorophores in individual pixels. The Pearson’s correlation coefficient is a well-established measure of correlation (Manders et al., 1992; Zinchuk and Grossenbacher-Zinchuk, 2011) and has range of +1 (perfect correlation) to −1 (perfect but negative correlation) with 0 denoting the absence of a relationship. The quantitative analysis was performed by using MATLAB^®^ Software.

### Western Blot Analysis

Five-millimeter-long segments from CHL1^−/−^ and CHL1^+/+^ mice spinal cord were dissected at the thoracic level. Samples were mechanically dissociated and lysed in radioimmunoprecipitation assay (RIPA) buffer (Sigma). Samples were briefly sonicated, heated at 96°C for 5 min, and centrifuged (20,600× *g*) at 4°C for 5 min. The supernatants were electrophoretically separated by SDS-PAGE on 4%–12% NuPAGE Novex Bis-Tris gradient gels (Invitrogen) and then transferred to nitrocellulose membranes (Invitrogen). Blots were incubated overnight at 4°C with primary antibodies (Table 1) and washed three times in Tris-buffered saline with 0.05% Tween 20 before probing with horseradish peroxidase-conjugated secondary antibodies (Jackson ImmunoResearch, West Grove, PA, USA) for 1 h at RT. Proteins of interest were then visualized with an enhanced chemiluminescence reagent (GE Healthcare, Piscataway, NJ, USA) and quantified with a laser-scanning densitometer (Canon, Lake Success, NY, USA).

## Results

### Ablation of CHL1 Does Not Influence the Locomotor Recovery After Femoral Nerve Injury

**Figure 1:**
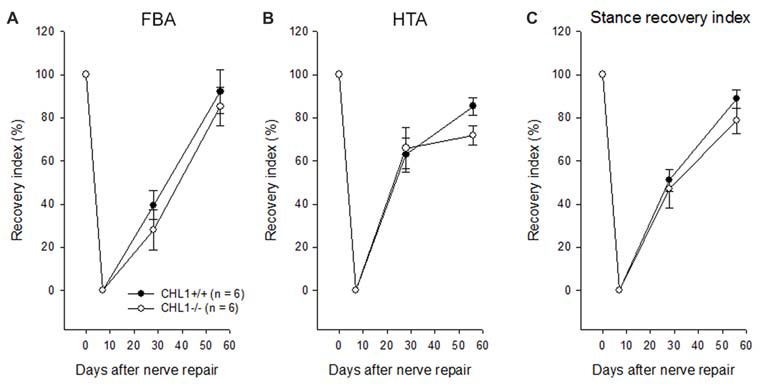
Time-course of motor recovery after femoral nerve lesion. Mean values ± SEM of recovery indices were estimated by measurements of foot-base angles (FBAs) **(A)** and heels-tail angles (HTAs) **(B)** 1 to 8 weeks after femoral nerve transection in CHL1^−/−^ and CHL1^+/+^ mice. Pre-operative values are plotted at day 0. **(C)** Individual animal values of the stance recovery index (RI) calculated using HTA and FBA indices (see “Materials and Methods” section for details). A RI of 100% indicates complete recovery. No significant difference between the genotypes was found at any time point (*p* > 0.05, one-way analysis of variance (ANOVA) for repeated measurements).

Experimental damage of the femoral nerve induced changes in the heels-tail (HTA) and the FBAs leading to a decrease in their RI to “0” being similar in CHL1^−/−^ and CHL1^+/+^ mice (Figures 1A,B). These alterations were caused in both genotypes by impaired extensor function of the quadriceps muscle, leading to abnormally high heel position at contralateral swing and external rotation at stance. The HTA and the FBA in mice of both genotypes gradually increased and approached normal values after 8 weeks, although the recovery remained incomplete in both groups of mice when compared to values in non-injured mice. A tendency in behavioral differences between genotypes in accordance with the Stance RI (mean of the RI for the HTA and the FBA) was observed 8 weeks after injury (Figure 1C, *p* > 0.05).

### CHL1^−/−^ Mice Display Impaired Regeneration of Motor Neurons in Quadriceps Branches

**Figure 2:**
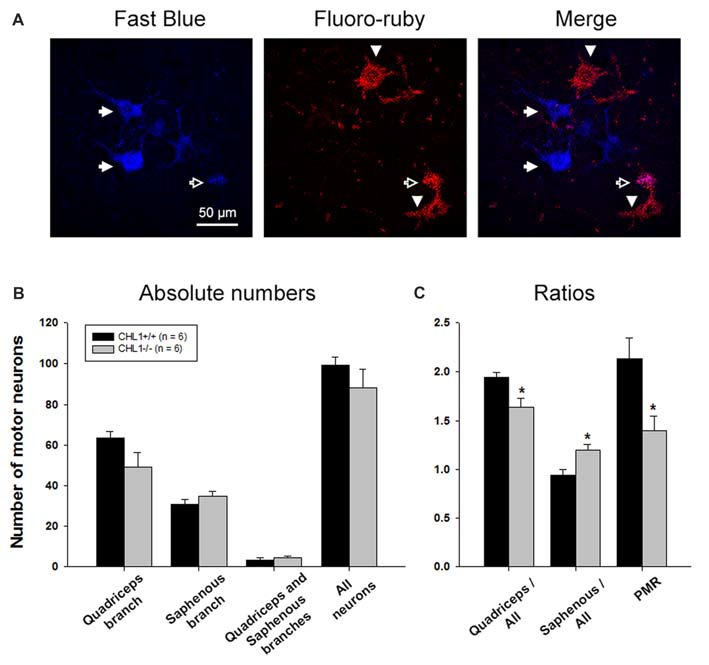
The number of motor neurons with axons regenerating into the quadriceps branch is reduced in CHL1^−/−^ mice. **(A)** Representative images of motoneurons labeled with Fast Blue (blue, correctly regrown into the quadriceps branch, shown with filled arrows), Fluoro-ruby (red, incorrectly regrown into the saphenous branch, shown with arrowheads), and merge (violet, regrown into both branches, quadriceps and saphenous, shown with open arrow). **(B)** Mean number of motor neurons (+SEM) labeled through either only the quadriceps or saphenous branches, through both branches, and the sum of neurons in the first three categories (“All neurons”) 8 weeks after femoral nerve injury in CHL1^−/−^ and CHL1^+/+^ mice. **(C)** Ratios between numbers of motor neurons correctly regenerated via quadriceps branches and motor neurons incorrectly regenerated via saphenous branches to the whole number of regenerated fibers. PMR—preferential motor re-innervation (number of motor neurons regenerated via quadriceps branches to number of motor neurons regenerated via saphenous branches). Asterisks indicate differences between CHL1^−/−^ and CHL1^+/+^ mice (*p* < 0.05, two-sided *t*-test for independent samples).

At 8 weeks after injury, the total number of motor neurons retrogradely labeled through either only the quadriceps or the saphenous branch, or through both branches of the femoral nerve (Figure 2A), did not significantly differ between CHL1^−/−^ and CHL1^+/+^ mice (Figure 2B). However, it is noteworthy that CHL1^−/−^ mice showed decreased preferential motor re-innervation (PMR, Figure 2C), i.e., the ability of motor neurons to regrow their axons more frequently into the appropriate quadriceps rather than into the inappropriate saphenous nerve branch (Brushart, 1988). The degree of PMR, estimated as an index of PMR (number of motor neurons projecting into the quadriceps branch/number of motor neurons projecting into the saphenous branch), was significantly higher in CHL1^+/+^ mice compared with CHL1^−/−^ mice. These results show reduced precision of motor re-innervation in CHL1^−/−^ mice.

### CHL1 Deficiency Enhances Axonal Regrowth/Sprouting After Femoral Nerve Injury

**Figure 3:**
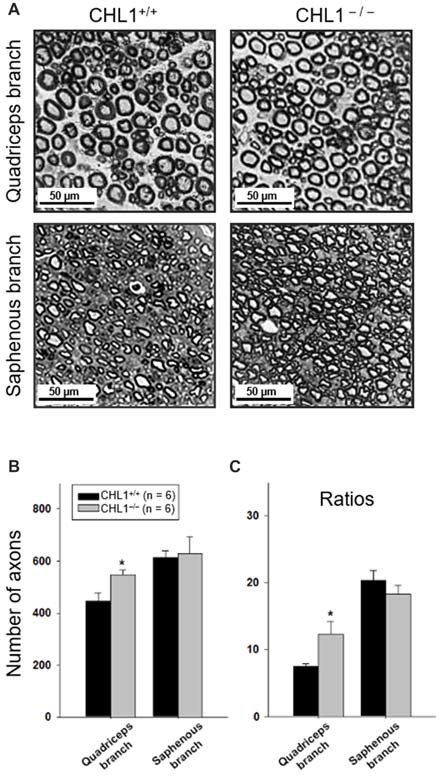
Regrowth/sprouting of regenerating axons is increased in CHL1^−/−^ mice. **(A)** Representative images of the distal part of quadriceps and saphenous nerve branches from CHL1^+/+^ and CHL1^−/−^ mice 8 weeks after femoral nerve injury. **(B)** Analysis of myelinated axons in regenerated quadriceps and saphenous nerve branches performed at 8 weeks after injury in CHL1^−/−^ and CHL1^+/+^ mice. **(C)** Ratios between numbers of regenerated axons and numbers of motor neurons calculated from individual animal values. Both panels **(B,C)** show mean values + SEM. Asterisks indicate differences between CHL1^−/−^ and CHL1^+/+^ mice (*p* < 0.05, *t*-test).

We next performed a morphological analysis of non-injured and regenerated nerves to evaluate the numbers of myelinated axons (Figure 3A). The axonal numbers in the regenerated quadriceps nerve branch were significantly higher in CHL1^−/−^ vs. CHL1^+/+^ mice (Figure 3B). Moreover, CHL1^−/−^ mice also showed a higher ratio of myelinated axons in the quadriceps nerve branch when compared to CHL1^+/+^ mice, indicating an increased axonal regrowth/sprouting in CHL1^−/−^ mice (Figure 3C).

### Inhibitory Synaptic Coverage in Non-injured CHL1^−/−^ Motor Neurons Is Increased, While Excitatory Synaptic Coverage in Non-injured and Regenerated CHL1^−/−^ Motor Neurons Is Reduced

**Figure 4:**
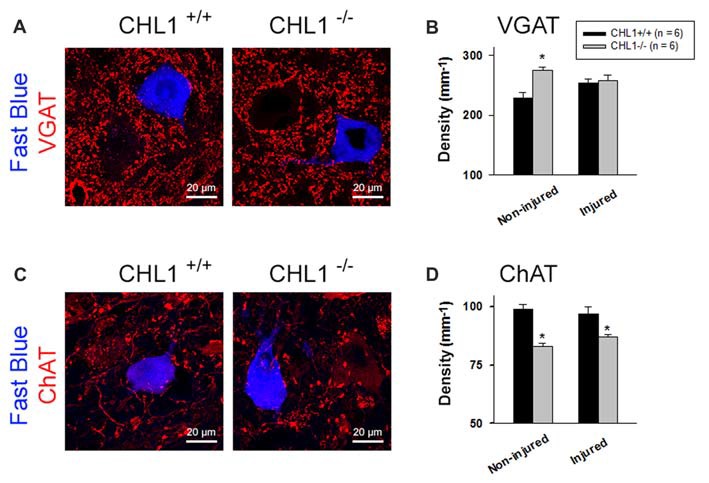
Analysis of perisomatic nerve terminals. **(A,C)** Representative pictures of vesicular GABA transporter (VGAT)- **(A)** and choline acetyltransferase (ChAT)-positive puncta **(C)** surrounding motor neurons retrogradely labeled with Fast Blue 8 weeks after femoral nerve transection. Perisomatic terminal densities were estimated by counting numbers of immunolabeled terminals adjacent to correctly projecting motor neuronal cell bodies (**A,C**, retrogradely labeled with Fast Blue, but not with Fluoro-ruby, applied to the quadriceps or saphenous nerve branch, respectively) and normalizing them to the soma perimeter. **(B,D)** Densities of VGAT^+^ **(B)** and ChAT^+^ **(D)** puncta in non-injured mice (subjected to retrograde tracing only) and mice studied 8 weeks after nerve repair (“Injured”). Graphs show group mean values + SEM calculated from individual mean values. Between 200 cells and 350 cells from six animals were analyzed per group and parameter. Asterisks in **(B,D)** indicate differences between CHL1^+/+^ and CHL1^−/−^ mice (*p* < 0.05, *t*-test).

Perisomatic synapses on motor neurons are known to be reduced in number after the damage to the axons. This phenomenon is also called “synaptic stripping” (Blinzinger and Kreutzberg, 1968; Moran and Graeber, 2004). When re-innervation is completed, motor neuron cell bodies regain synaptic input to varying degrees (Brannstrom and Kellerth, 1999). To elucidate the role of CHL1 during this process, we estimated the linear density of perisomatic puncta immunoreactive for the VGAT and cholinergic marker ChAT around retrogradely labeled motor neuron somata at 8 weeks after injury (Simova et al., 2006; Figures 4A,C). These neurons express CHL1 (Supplementary Figure S1A). Cholinergic synapses were selected for analysis for three main reasons: (1) coverage of motor neuronal cell bodies with C-type synapses is strongly reduced after peripheral nerve lesion, and restitution after regeneration is incomplete (Brannstrom and Kellerth, 1999; Hellström et al., 2003); (2) large cholinergic nerve terminals form synapses with postsynaptic cisterns (C-type) on motor neuron cell bodies containing muscarinic type 2 receptors (Davidoff and Irintchev, 1986; Hellström et al., 2003), voltage-gated K^+^ channels Kv2.1 (Muennich and Fyffe, 2004), Ca^2+^-activated K^+^ (SK) channels (Deardorff et al., 2013), vesicle-associated membrane protein 2 (VAMP-2; Hellström et al., 1999), and sigma-1 receptors (S1Rs; Mavlyutov et al., 2013), known to modulate motor neuronal activity during locomotion (Miles et al., 2007; Zagoraiou et al., 2009); and (3) large and intensely labeled ChAT-positive boutons surrounding motor neuron perikarya are clearly discernible, and thus readily quantifiable (Simova et al., 2006).

Analysis of inhibitory (GABAergic and glycinergic, VGAT^+^) terminals and modulatory cholinergic (ChAT^+^) terminals around motor neuron perikarya revealed differences between the CHL1^+/+^ and CHL1^−/−^ mice without injury (“Non-injured” groups in Figure 4). The density (number per unit length of the surface membrane of a perikaryon) of VGAT^+^ terminals was higher (Figures 4A,B), and that of ChAT^+^ puncta was lower (Figures 4C,D) in CHL1^−/−^ animals as compared to CHL1^+/+^ mice. After the nerve injury, densities of ChAT^+^ terminals (“Injured” groups in Figure 4D) in both genotypes were not changed, when compared with the respective non-injured groups. The density of ChAT^+^ terminals after injury remained lower in CHL1^−/−^ vs. CHL1^+/+^ mice (Figure 4D). The densities of VGAT^+^ boutons were not different in injured vs. non-injured animals of both genotypes (Figure 4B) and were also not different in injured CHL1^−/−^ vs. CHL1^+/+^ mice (Figure 4B). The density of VGAT^+^ boutons was higher in non-injured CHL1^−/−^ vs. CHL1^+/+^ mice. After nerve injury, the density of VGAT^+^ boutons on motor neurons was slightly increased in CHL1^+/+^ mice and declined in CHL1^−/−^ mice, suggesting different post-traumatic synaptic rearrangements on motor neurons in CHL1^−/−^ and CHL1^+/+^ mice. Thus, CHL1^−/−^ mice revealed increased numbers of inhibitory synapses, but decreased numbers of cholinergic synapses, independently of femoral nerve injury.

### CHL1 Expression After Nerve Damage

**Figure 5:**
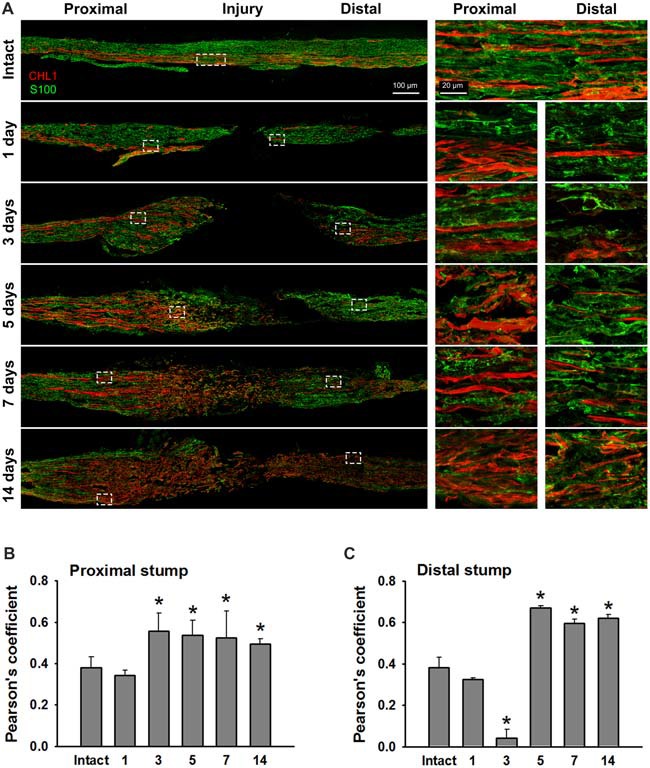
Immunofluorescence analysis of CHL1 expression in Schwann cells in non-injured and injured femoral nerves. **(A)** Longitudinal cryosections from a non-injured nerve (“Intact”) and from femoral nerves of CHL1^+/+^ mice dissected 1, 3, 5, 7 and 14 days after injury were analyzed with antibodies against CHL1 (red) and the Schwann cell marker S100 (green). **(B,C)** Quantitative analysis of co-localization of CHL1 and S100 identified by Pearson’s coefficient, where “+1” is a perfect correlation, “0”—no correlation, and “−1”—perfect anti-correlation. Graphs show mean values + SEM (*p < 0.01 compared with non-injured nerve).

**Figure 6:**
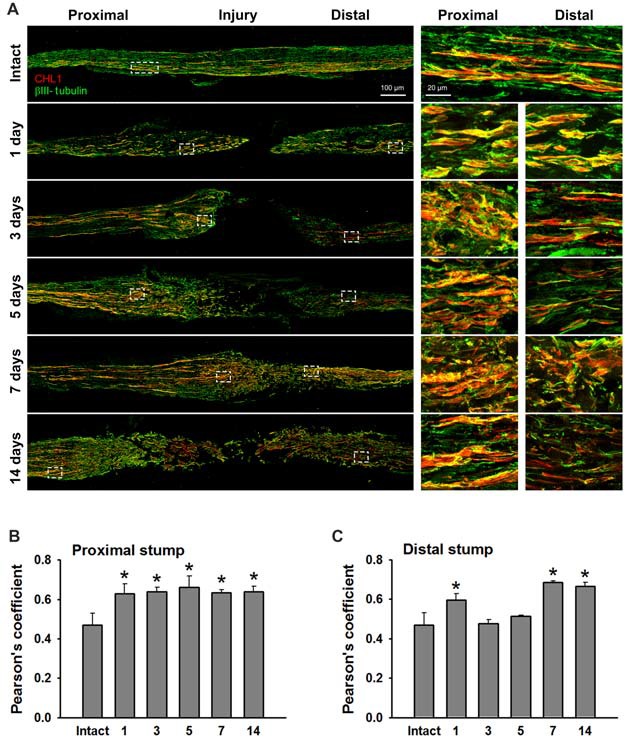
CHL1 expression in the femoral nerve after injury is mainly detected in regrown axons. **(A)** Longitudinal cryostat sections from intact and injured femoral nerves of CHL1^+/+^ mice at 1, 3, 5, 7 and 14 days after nerve transection. Immunofluorescence analysis with antibodies against CHL1 (red) and the neuronal marker βIII-tubulin (green). **(B,C)** Quantitative analysis of co-localization of CHL1 and βIII-tubulin identified by Pearson’s coefficient, where “+1” is a perfect correlation, “0”–no correlation, and “−1”–perfect anti-correlation. Graphs show mean values + SEM (*p < 0.01 compared with non-injured nerve).

We analyzed sections from femoral nerves at 1, 3, 5, 7 and 14 days after injury in CHL1^+/+^ mice using the antibody against mouse CHL1 and antibodies against the Schwann cell marker S100 (Figure 5A), or the neuronal βIII-tubulin marker (Figure 6A). Specificity of the anti-CHL1 antibody was confirmed by immunofluorescence labeling of spinal cord and femoral nerve cryosections as well as Western blot analysis of spinal cord tissue from CHL1^+/+^ and CHL1^−/−^ mice (Supplementary Figures S1A–D). Quantitative analysis using Pearson’s coefficient showed higher CHL1 expression in regenerating axons of the injured femoral nerve (Figures 6B,C) as compared to CHL1 expression in Schwann cells (Figures 5B,C) in both the proximal and distal nerve stumps. Both, regenerating motor neurons and DRG neurons expressed CHL1 (Supplementary Figures S1A,E). Pearson’s coefficients for CHL1 and βIII-tubulin vs. CHL1 and S100 co-localization in proximal stumps at days 1, 3, 5, 7 and 14 after injury were 0.6, 0.6, 0.7, 0.6 and 0.6 vs. 0.3, 0.5, 0.5, 0.5 and 0.5, respectively. Interestingly, expression of CHL1 in Schwann cells was increased in the proximal stump after injury compared to the non-injured nerve, as first observed only at day 3 after injury, being relatively less pronounced compared to axonal CHL1 expression. In contrast, CHL1 expression in injured axons was increased already 1 day after injury. It is important to note that Schwann cells increase CHL1 expression between days 3 and 5 after injury, suggesting that at this stage CHL1 supports the guidance of the regrowing axons, but suppresses axonal regrowth/sprouting, most likely due to homophilic interaction between axons and Schwann cells.

## Discussion

**Figure 7:**
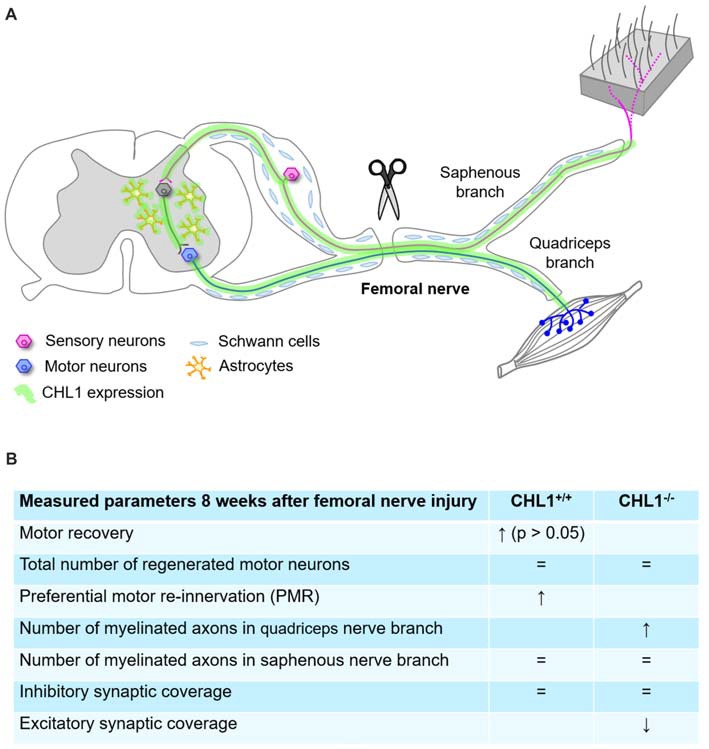
Summary diagram representing schematically the femoral nerve injury and CHL1 expression in CNS and peripheral nervous system (PNS) **(A)**, and parameters measured 8 weeks after femoral nerve injury **(B)**, where “=” means no difference. ↑: expression up, ↓: expression down.

We have reported that CHL1 deficiency results in improved motor recovery after spinal cord injury (Jakovcevski et al., 2007). The improved recovery correlates with enhanced monoaminergic re-innervation of the spinal cord caudal to the injury site, as reflected by increased numbers of axons below the injury site in CHL1^−/−^ mice. In contrast to these findings in the CNS, results of the present study indicate that CHL1 has a tendency to support the recovery of motor functions after the femoral nerve lesion, which is, however, statistically not significant. Earlier studies indicated that after injuries of the adult mouse CNS or PNS, CHL1 is upregulated in its expression in both neurons and astrocytes. The basic fibroblast growth factor (FGF-2)-dependent upregulation of CHL1 expression in astrocytes of the scar contributes to the negative impact of CHL1 on spinal cord regeneration, where homophilic binding of CHL1 on axons and astrocytes inhibits neurite outgrowth (Jakovcevski et al., 2007). Here we show that the upregulation of CHL1 expression in injured peripheral nerves already at 1 day after injury is detectable in the regrowing axons but not in Schwann cells, suggesting that only heterophilic interactions between CHL1 on growing axons and yet unknown receptors on other cells are activated (Figure 7). Impaired targeted regeneration of motor neurons into the quadriceps nerve branch in CHL1^−/−^ mice, being accompanied by a slight reduction in motor recovery in CHL1^−/−^ mice, suggests that heterophilic interactions of CHL1 in CHL1^+/+^ mice can have a favorable effect on axonal guidance during early stages of regeneration. Since previous studies indicated that CHL1 deficiency is associated with upregulation of the NCAM 180 (Montag-Sallaz et al., 2002), it is conceivable that expression of other cell adhesion molecules might be also dysregulated, thereby compensating for the loss of CHL1.

Despite the lack of genotype-related differences in functional recovery, numbers of myelinated axons in the regenerated quadriceps branch of the femoral nerve in CHL1^−/−^ were increased by approximately 20%, indicating that regrowth/sprouting of axons is increased in CHL1^−/−^ mice. Increased regrowth of axons after the spinal cord injury under conditions of CHL1 deficiency has been attributed to the absence of the homophilic CHL1 interactions, which inhibit axonal growth (Jakovcevski et al., 2007; Wu et al., 2010; Katic et al., 2014). CHL1 is strongly expressed in the PNS and, in particular, in femoral and sciatic nerves of newborn mice (Hillenbrand et al., 1999). In adult non-injured mice, CHL1 mRNA is expressed at moderate levels by small- to medium-sized sensory neurons and non-myelinating Schwann cells in the sensory branches of the peripheral nerve, whereas only little or no CHL1 mRNA is expressed in motor neurons (Hillenbrand et al., 1999; Zhang et al., 2000). Similar to the increase in CHL1 expression after spinal cord injury (Jakovcevski et al., 2007), CHL1 expression increases after injury in the PNS, e.g., in motor neurons and small- to medium-sized sensory neurons after sciatic nerve injury (Zhang et al., 2000; Barrette et al., 2010). In the present study, CHL1 expression was found to be also increased in regenerating axons of lesioned femoral nerves. Notably, a high increase in CHL1 expression was detected in Schwann cells as early as 3 and 5 days after injury. These observations suggest that heterophilic interactions of CHL1 expressed by regenerating motor neurons facilitate axonal regrowth during the first 2 days after femoral nerve injury and could explain the higher degree of PMR in CHL1^+/+^ mice compared with CHL1^−/−^ mice.

The inhibitory effect of CHL1 on axonal regrowth through the spinal cord injury site has also been linked to the induction of CHL1 expression in the scar-forming glial cells with homophilic interactions between CHL1 on growing axons and CHL1 on glial cells, contributing to inhibition of axonal growth (Jakovcevski et al., 2007). CHL1 is expressed in primary cultures of S100-positive Schwann cells and in cultures of the Schwann cell line S-16 (Hillenbrand et al., 1999). Increased levels of CHL1 mRNA in Schwann cells was reported after sciatic nerve injury (Zhang et al., 2000). By comparing the effects of CHL1 deficiency in the spinal cord with that in the lesioned femoral nerve it is interesting that in the femoral nerve only a 20% increase in CHL1 expression was found compared to the pronounced, 2-fold increase, in the injured spinal cord (Jakovcevski et al., 2007).

While the numbers of regrown/sprouted axons in the quadriceps branch are increased in injured CHL1-deficient femoral nerves, the precision of preferential motor re-innervation after femoral nerve injury is reduced in CHL1^−/−^ mice, suggesting that CHL1 is involved in regulating the guidance of motor neuron axons into the quadriceps vs. the saphenous branch. CHL1 expression in motor neurons is upregulated in response to injury (Zhang et al., 2000), and this may contribute to the specificity reflected by preferential motor re-innervation. Semaphorin 3A and neuropilin 1/2 signaling controls distinct steps of motor axon growth and guidance not only in the development of spinal motor connections (Huber et al., 2005), but also in regeneration of the PNS and CNS after injury (Pasterkamp and Verhaagen, 2006). Since CHL1 deficiency results in impaired responsiveness to semaphorin 3A (Wright et al., 2007; Schlatter et al., 2008), it is possible that a decline in the precision of preferential motor re-innervation is linked to a defect in semaphorin 3A signaling, a possibility that awaits investigation in the future.

Previous studies indicated that CHL1 regulates the GABAergic synaptic connectivity in the brain. Numbers of inhibitory interneurons are increased in the hippocampus and cingulate cortex of juvenile CHL1^−/−^ mice (Nikonenko et al., 2006; Schmalbach et al., 2015) and this increase correlates with the increased density of perisomatic inhibitory synapses in the hippocampus of juvenile CHL1^−/−^ mice (Nikonenko et al., 2006). The numbers of inhibitory interneurons decline with age in the hippocampus of CHL1^−/−^ mice but remain increased in the cingulate cortex of 2- to 6-month-old CHL1^−/−^ mice (Schmalbach et al., 2015), i.e., at the age analyzed in the present study. Our observation that inhibitory perisomatic innervation of motor neurons analyzed by retrograde labeling and immunohistochemistry in spinal cords of CHL1^−/−^ mice is increased compared to their CHL1^+/+^ littermates is in agreement with our previous investigation (Nikonenko et al., 2006) showing that in juvenile (3- to 4-week-old) CHL1^−/−^ mice, the perisomatic inhibitory input to CA1 pyramidal neurons is increased. Noteworthy, these animals display increased inhibitory postsynaptic currents evoked in pyramidal cells by minimal stimulation of perisomatically projecting interneurons and reduced long-term potentiation (LTP) at CA3-CA1 excitatory synapses (Nikonenko et al., 2006). We can expect, therefore, that motor neurons of CHL1^−/−^ mice would have a reduced spiking (action potential) activity and decreased spontaneous excitatory post-synaptic potentials.

Whether CHL1 is involved in regulating cholinergic synapse formation and/or numbers of cholinergic interneurons remains to be investigated.

## Conclusion

Our data indicate that CHL1 is involved in peripheral nerve regeneration by guidance of regenerating axons to yield preferential motor re-innervation, controlling synaptic circuitry in the spinal cord and regulating axonal regrowth/sprouting at early stages of peripheral nerve regeneration via heterophilic interactions.

## Author Contributions

DG, IJ, AI and MS: study concept and design. DG, IJ and AI: acquisition of data and statistical analysis. DG, IJ, AI, IL, VS, EP and MS: drafting of the manuscript and technical support. DG, EP and MS: obtained funding. MS: study supervision.

## Conflict of Interest Statement

The authors declare that the research was conducted in the absence of any commercial or financial relationships that could be construed as a potential conflict of interest.

## Supplementary Material

FIGURE S1 Expression of the close homolog of L1 (CHL1) in motor and DRG neurons as well as in femoral nerves. **(A,B)** Representative images of the immunofluorescence analysis of spinal cord cryosections with anti-CHL1 antibody in CHL1^+/+^ **(A)** and CHL1^−/−^ **(B)** mice. Motor neurons retrogradely labeled with Fast Blue 8 weeks after femoral nerve transection are shown in blue. White arrows indicate CHL1-expressing neuron. **(C)** Representative images of the immunofluorescence analysis of femoral nerve cryosections with anti-CHL1 antibody in CHL1^+/+^ and CHL1^−/−^ mice. **(D)** Western blot analysis of spinal cord lysates from CHL1^+/+^ and CHL1^−/−^ mice. Upper panel—CHL1, lower panel—L1 as a loading control. **(E)** Representative images demonstrating CHL1 expression in DRG in non-injured CHL1^+/+^ mice.
