# Supplementary figures and images for: Cell Adhesion Molecule Close Homolog of L1 (CHL1) Guides the Regrowth of Regenerating Motor Axons and Regulates Synaptic Coverage of Motor Neurons
Source: Front Mol Neurosci. 2018 May 24;11:174. doi: 10.3389/fnmol.2018.00174 (PMC5976800; doi:10.3389/fnmol.2018.00174)

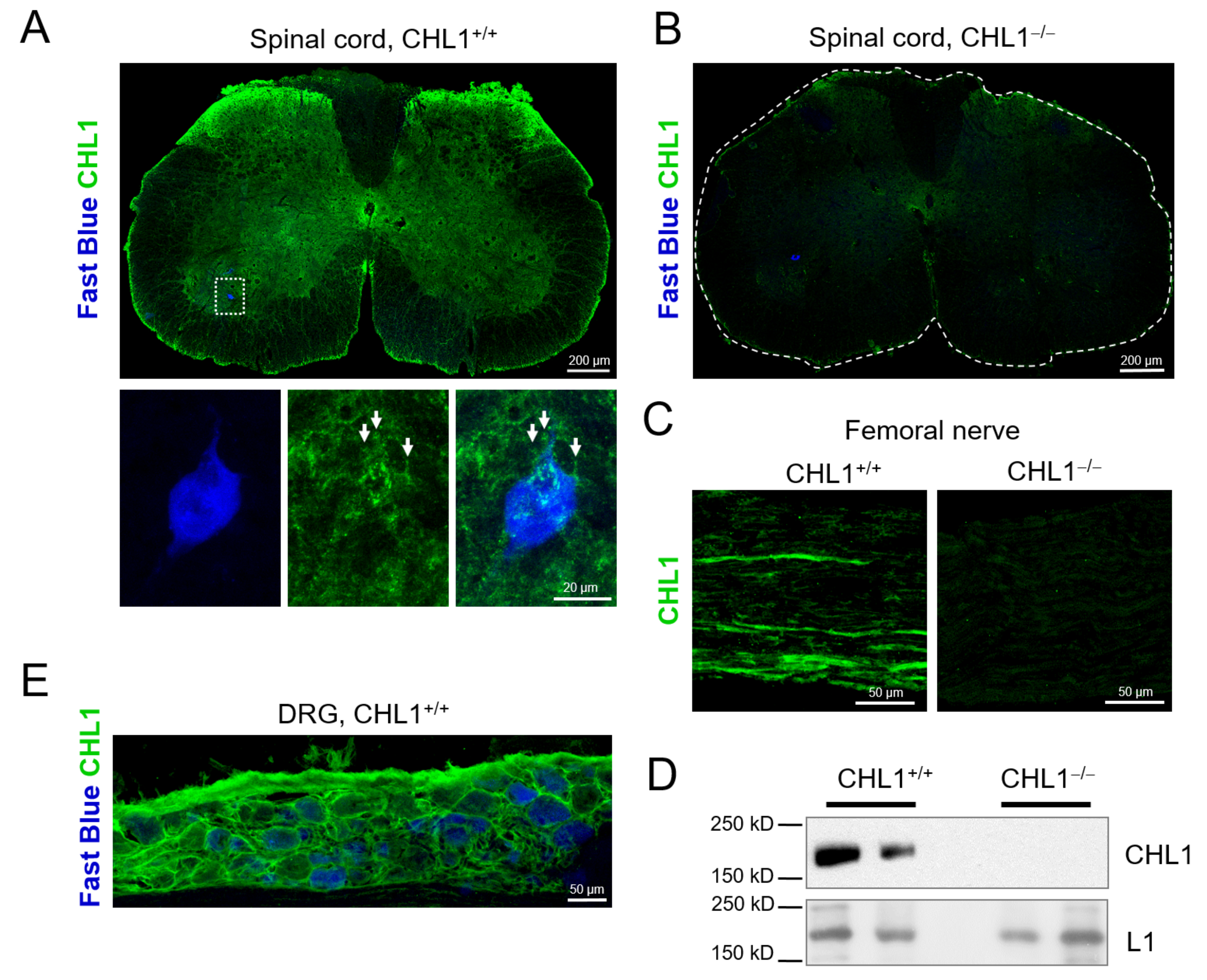

Supplement: FIGURE S1 — Expression of the close homolog of L1 (CHL1) in motor and DRG neurons as well as in femoral nerves. (A,B) Representative images of the immunofluorescence analysis of spinal cord cryosections with anti-CHL1 antibody in CHL1+/+ (A) and CHL1−/− (B) mice. Motor neurons retrogradely labeled with Fast Blue 8 weeks after femoral nerve transection are shown in blue. White arrows indicate CHL1-expressing neuron. (C) Representative images of the immunofluorescence analysis of femoral nerve cryosections with anti-CHL1 antibody in CHL1+/+ and CHL1−/− mice. (D) Western blot analysis of spinal cord lysates from CHL1+/+ and CHL1−/− mice. Upper panel—CHL1, lower panel—L1 as a loading control. (E) Representative images demonstrating CHL1 expression in DRG in non-injured CHL1+/+ mice. [file Image_1.TIF]
